# Coagulopathy after hemorrhagic traumatic brain injury, an observational study of the incidence and prognosis

**DOI:** 10.1007/s00701-019-04111-z

**Published:** 2019-11-18

**Authors:** Jort A. N. van Gent, Thomas A. van Essen, Mettine H. A. Bos, Suzanne C. Cannegieter, Jeroen T. J. M. van Dijck, Wilco C. Peul

**Affiliations:** 1 Department of Neurosurgery, Leiden University Medical Center, University Neurosurgical Center Holland (UNCH), Albinusdreef 2, 2333 ZA Leiden, The Netherlands; 2 HAGA Teaching Hospital, The Hague, The Netherlands; 3 Haaglanden Medical Center, The Hague, The Netherlands; 4 Division of Thrombosis and Hemostasis, Leiden University Medical Center, Leiden, The Netherlands; 5 Department of Clinical Epidemiology, Leiden University Medical Center, Leiden, The Netherlands

**Keywords:** Traumatic brain injury, Coagulopathy, Progression of hemorrhagic injury, Time course, Mortality

## Abstract

**Background:**

Traumatic brain injury is associated with high rates of mortality and morbidity. Trauma patients with a coagulopathy have a 10-fold increased mortality risk compared to patients without a coagulopathy. The aim of this study was to identify the incidence of coagulopathy and relate early coagulopathy to clinical outcome in patients with traumatic intracranial hemorrhages.

**Methods:**

Between September 2015 and December 2016, 108 consecutive cranial trauma patients with traumatic intracranial hemorrhages were included in this study. To assess the relationship between patients with a coagulopathy and outcome, a chi-squared test was performed.

**Results:**

A total of 29 out of the 108 patients (27%) with a traumatic intracranial hemorrhage developed a coagulopathy within 72 h after admission. Overall, a total of 22 patients (20%) died after admission of which ten were coagulopathic at emergency department presentation. Early coagulopathy in patients with traumatic brain injury is associated with progression of hemorrhagic injury (odds ratio 2.4 (95% confidence interval 0.8–8.0)), surgical intervention (odds ratio 2.8 (95% confidence interval 0.87–9.35)), and increased in-hospital mortality (odds ratio 23.06 (95% confidence interval 5.5–95.9)).

**Conclusion:**

Patients who sustained a traumatic intracranial hemorrhage remained at risk for developing a coagulopathy until 72 h after trauma. Patients who developed a coagulopathy had a worse clinical outcome than patients who did not develop a coagulopathy.

## Introduction

Traumatic brain injury (TBI) is a major cause of mortality and disability globally, and unfortunately, mortality and neurological morbidity has not decreased substantially over the last 30 years [9, 12, 28]. One of the most significant parameters that influences patient outcome is the presence of a traumatic intracranial hemorrhage (t-ICH). Patients sustaining moderate and severe TBI (i.e., a Glasgow Coma Scale (GCS) between 3 and 12) often have intracranial bleeding such as an acute subdural hemorrhage (ASDH), an epidural hemorrhage (EDH), a cerebral contusion (CC), a subarachnoid hemorrhage (SAH) or a combination of them, which can be accompanied by secondary injury. This secondary injury may consist of brain swelling and progression of hemorrhagic injury (PHI) which, ultimately, could form a risk of herniation [12, 23]. For the focal lesions ASDH, EDH, and/ or contusions, surgical evacuation is the cornerstone of treatment. Creating optimal conditions for surgical treatment and prevention of PHI can have a major impact on mortality and morbidity [4]. One of these conditions is a proper hemostasis. Unfortunately, after TBI, hemostasis is often derailed, leading to a hypocoagulopathic and—less often—a hypercoagulopathic state [17]. Multiple studies have found that one third of all patients who sustained a TBI developed a coagulopathy due to trauma [8]. Other studies related such a trauma-induced coagulopathy with PHI and found it to be a powerful independent risk factor for mortality [1, 7, 11, 25]. Patients with a coagulopathy after TBI have a 10-fold increased mortality risk in addition to a 30-fold higher risk of unfavorable outcome [8, 17].

While the pathophysiological mechanisms are largely unknown, one of the most likely hypothesis is that after a TBI, the naturally occurring abundance of tissue factor (TF) in the microvessels of the brain is massively released into the bloodstream [13]. This triggers the coagulation cascade via the external coagulation pathway leading to consumptive insufficiency of coagulation factors [14]. Another leading (additional) hypothesis is that a combination between shock and hypoperfusion results in activation of protein C [5, 21], which promotes fibrinolysis through inactivation of plasminogen activator inhibitor-1 [27]. These hypotheses are mainly theorized and based on scarce empirical evidence.

Thus, there is a lack of insight into TBI-associated coagulopathies that sharply contrasts with their potential clinical consequences. First and foremost, it is unknown whether or not the coagulopathy is associated with intracranial hemorrhage after trauma. This multicenter cohort study aims to describe the incidence and time course of coagulopathy in patients with t-ICH. Additionally, the relation between TBI-associated coagulopathy and outcome will be explored.

## Material and methods

### Study population

Consecutive adult patients with TBI after isolated blunt head trauma between September 2015 and December 2016 were identified from the emergency department (ED) registry in three cooperating level 1 trauma centers within the University Neurosurgical Center Holland (Leiden University Medical Center, Haga Teaching Hospital, and Haaglanden Medical Centrum). In this recruitment period, the observational study CENTER-TBI was also active and did include the same patients as this study [24]. However, no data of the CENTER-TBI study was used. Patients were included when (1) they had suffered from TBI, (2) had presence of an intracranial hemorrhage (ASDH, EDH, SAH, CC) confirmed by the first posttrauma computed tomography (CT) scan, and (3) were primary presented to the participating hospital within 6 h after injury. Patients were excluded in case they (1) suffered from severe extracranial injuries (non-head Abbreviated Injury Score (AIS) > 3), (2) had no coagulation parameters available, (3) had a history of coagulation disorders, and (4) used anticoagulants. Medical Ethics Committee approval was granted (G16.087).

### Study parameters

The following data were collected: demographic baseline data, GCS at ED presentation, extracranial AIS, medical history categorized by the American Society of Anesthesiologists Physical status Classification system (ASA) [6], surgeries performed on the patient, ICU admission, length of stay, and mortality. The use of anticoagulants or antifibrinolytics during hospital stay was not considered because this information was not readily available. Patients who died during hospital admission were categorized as follows: died within 24 h after hospital admission, died between 24 and 48 h, died between 48 and 72 h, or died within 72 and 96 h and died after 96 h.

### Definition of coagulopathy

Patients were categorized into a coagulopathy and a no-coagulopathy group, based on the coagulation profile drawn at presentation to the ED. Coagulopathy was defined as an international normalized ratio (INR) > 1.20 and/or a platelet count below 150 × 10^3^/μl and/or an activated partial thromboplastin time (APTT) > 34.5 s [2, 31]. To investigate the incidence of coagulation, blood drawn after 24, 48, 72, and 96 h of admission was also included, if available.

### Cranial computed tomography

All evaluations of CT scans were extracted from the original report by the radiologist who was unaware of the study during the conduct of the report. PHI was defined as an increase in volume of the initial hemorrhage or development of new intracranial hemorrhages at sites distant from previous lesions, at the discretion of the attending radiologist. Patients who had no follow-up CT scan because they were clinically stable were classified as no PHI.

### Statistical analysis

The mean and standard deviation (SD) or median and interquartile range (IQR) were calculated according to distribution and scale of measurement. To assess the relationship between patients who developed coagulopathy at ED presentation and mortality or PHI, a chi-squared test was performed with mortality and PHI as outcome variables. For these outcome variables, an odds ratio (OR) with a 95% confidence interval (CI) was calculated. All analyses were performed using the Statistical Package for Social Sciences (SPSS 25.0).

## Results

### Subject recruitment

**Fig. 1:**
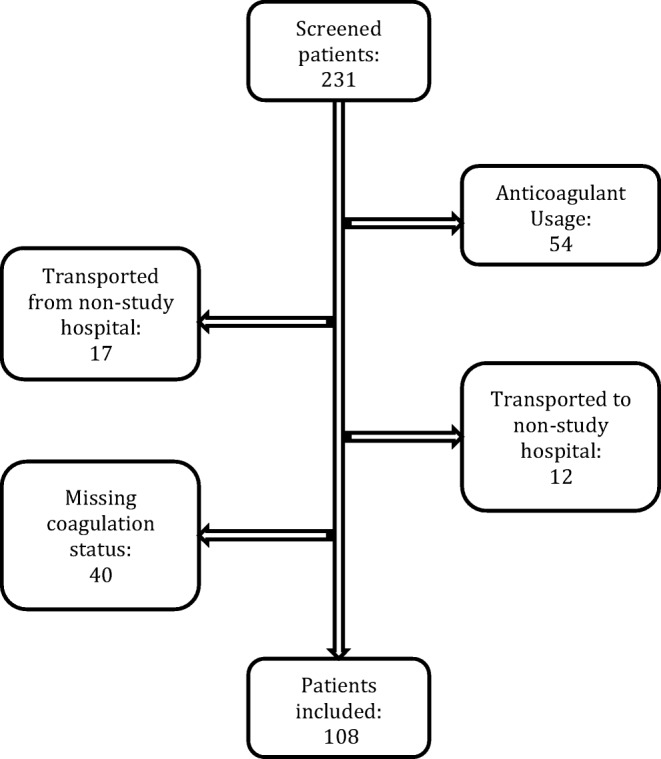
Flowchart

From a total of 231 potential study patients with a t-ICH, 108 were included. Fifty-four patients were excluded because of anticoagulant usage, 29 patients were excluded because of secondary referral, and forty patients were excluded because of missing coagulation status at the participating hospital (Fig. 1).

### Baseline characteristics

Median cohort age was 60 years (interquartile range (IQR) 31), and gender distribution was equal (Table 1). Based on the ASA physical status classification, only three patients (3%) had a severe systemic disease. Most patients were diagnosed with an ASDH (61%) followed by a SAH (59%), an CC (56%), and an EDH (16%). Sixty-nine patients (64%) presented with a skull fracture. Many patients (69%) sustained a combination of t-ICH. The most frequent combinations were ASDH + CC + SAH (17%) and ASDH + SAH (16%).

**Table 1:** Clinical characteristics of the study cohort, consisting of 108 patients who sustained an acute traumatic intracranial bleeding. Continuous variables denoted as means with standard deviation (SD), categorical data as counts (%). Median was used when there was non-normal distribution with interquartile range (IQR)

Characteristics	*n* (%)
Total patients	108 (100)
Age, median (IQR)	60 (31)
Male sex	55 (51)
ASA score	
Normal healthy person	67 (62)
Person with a mild systemic disease	38 (35)
Patient with a severe systemic disease	3 (3)
GCS on admission, median (IQR)	12 (8)
Type of lesion *	
ASDH	66 (61)
EDH	17 (16)
CC	60 (56)
SAH	64 (59)
Combination of hemorrhages	75 (69)
Extracranial AIS on admission, median (IQR)	2 (3)
Number of CT-scans performed, median (IQR)	2 (1)
Midline shift	31 (29)
Skull fracture	69 (64)
Progression of hemorrhagic injury	38 (35)
In-hospital mortality	22 (20)
Coagulation Parameters	
HB, mean (SD)	8.2 (1.1)
Platelets, mean (SD)	230.7 (67.7)
Hypertension	24 (22.2)
Duration of hospital stay, median (IQR)	8 (11)
Admitted to ICU	71 (66)
Patients operated	28
Operations performed	38
Evacuation of hemorrhage	10 (10)
Intracranial pressure monitoring device	9 (8)
Decompressive craniectomy	11 (10)
External ventricular drain	8 (7)

Some patients had multiple intracranial lesions

*ASA* American Society of Anesthesiologists, *GCS* Glasgow Coma Scale, *ASDH* acute subdural hemorrhage, *EDH* epidural hemorrhage, *CC* cerebral contusion, *SAH* subarachnoid hemorrhage, *AIS* Abbreviated Injury Scale, *CT scan* computed tomography scan, *HB* hemoglobin, *ICU* intensive care unit

Twenty-eight patients underwent a total of 38 surgical interventions. The most frequently performed procedure was a decompressive craniotomy (29%) followed by a craniotomy with evacuation of the hemorrhage (28%), placement of an intracranial pressure measurement device (24%), and placement of an external ventricular drain (21%).

### Coagulopathy at ED presentation vs. no coagulopathy

Of the 108 patients, thirteen patients (12%) showed a coagulopathy on ED presentation leaving 95 patients (88%) with undisturbed coagulation parameters at presentation to the ED. Baseline parameters, including age and AIS at arrival, were similar in the coagulopathy and no-coagulopathy groups (Table 2). Male preponderance was existent in the coagulopathy group compared to the no-coagulopathy group (77 vs. 47%). Patients in the coagulopathy group presented with a lower GCS (median (IQR) GCS of 5 (5) compared to 13 (6) in the no-coagulopathy group (*p* = 0.00)), which reflects the severity of the injury. The majority of patients in the coagulopathy and no-coagulopathy group had an ASA score of ≤ 2 before trauma (98% and 92%, respectively), which reflects the healthy status of patients in both groups. In the coagulopathy group, most patients sustained a combination of ASDH+CC+SAH (46%) followed by ASDH+SAH (23%) and the third most common intracranial hemorrhage was an ASDH (15%). In the no-coagulopathy group, most patients sustained a combination of ASDH+SAH (16%) followed by ASDH+CC+SAH (13%) and ASDH+CC (12%).

**Table 2:** Characteristics of no-coagulopathy and coagulopathy group. Continuous variables denoted as means with standard deviation (SD), or medians with interquartile range (IQR) and categorical data as proportions (%)

Characteristics	Non-coagulopathic patients, *n* (%)	Coagulopathic patients at admission, *n* (%)	*p* value
Total patients	95 (88)	13 (12)	
Age, median (IQR)	61 (31)	58 (35)	0.92
Male sex	45 (47)	10 (77)	0.05
ASA score			
A normal healthy person	62 (65)	5 (39)	0.06
Patient with a mild systemic disease	31 (33)	7 (53)	0.29
Patient with a severe systemic disease	2 (2)	1 (8)	0.25
GCS on admission, median (IQR)	13 (6)	5 (5)	0.00
Type of lesion*			
ASDH	55(58)	11 (85)	0.07
EDH	17 (18)	0 (0)	0.10
CC	52 (55)	8 (62)	0.65
SAH	54 (57)	10 (77)	0.17
Combination of hemorrhage	64 (67)	11 (85)	0.21
Extracranial AIS on admission, median (IQR)	2 (3)	0 (2)	0.14
CT-scans performed, median (IQR)	2 (1)	2 (2)	0.55
Midline shift	23 (24)	8 (62)	0.01
Skull fracture	59 (62)	10 (77)	0.30
Progression of hemorrhagic injury	31 (33)	7 (54)	0.10
In-hospital mortality	12 (13)	10 (77)	0.00
HB, mean (SD)	8.3 (0.9)	7.3 (1.9)	0.00
Platelets, mean (SD)	239 (49.9)	170 (127.5)	0.00
Hypertension	23 (24.2)	1 (7.7)	0.15
Duration of hospital stay, median (IQR)	8 (9)	3 (14)	0.52
Admitted to ICU	59 (62)	12 (92)	0.03
Patients operated	22 (23)	6 (43)	0.16
Operation performed	29 (31)	8 (62)	0.08
Evacuation of hemorrhage	8 (8)	2 (15)	0.42
Intracranial pressure monitoring device	7 (6)	3 (23)	0.10
Decompressive craniectomy	7 (6)	3 (23)	0.10
External ventricular drain	8 (8)	0 (0)	0.28

Coagulopathy was defined as an international normalized ratio (INR) > 1.2 and/or a platelet count below 150 × 10^3^/μl and/or activated partial thromboplastin time (APTT) > 34.5 s

*ASA* American Society of Anesthesiologists, *GCS* Glasgow Coma Scale, *ED* emergency department, *ASDH* acute subdural hemorrhage, *EDH* epidural hemorrhage, *CC* Cerebral Contusion, *SAH* subarachnoid hemorrhage, *AIS* Abbreviated Injury Scale, *CT scan* computed tomography scan, *HB* hemoglobin, *ICU* intensive care unit, *ICP* intracranial pressure

The proportion of patients that required surgery was twice as large in the coagulopathy group relative to the no-coagulopathy group (46% vs. 23%) with an OR of 2.8 (95% CI 0.87–9.35), and patients in the coagulopathy group were more likely to be admitted to the ICU than patients in the no-coagulopathy group (92% vs. 62%) with an OR of 7.3 (95% CI 0.91–58.71).

The median number of CT scans taken during admission was similar for both groups being two per patient. There was a higher proportion of skull fractures in the coagulopathy group than in the no-coagulopathy group (77% vs. 62%) with an OR of 2.0 (95% CI 0.53–7.87). Patients with coagulopathy presented with a higher rate (62%) of midline shift compared to the no-coagulopathy group (62 vs. 24%) with an OR of 5.0 (1.49–16.83). Overall, a total of 22 patients (20%) died. CT scan analysis demonstrated that a total of 38 patients (35%) suffered from PHI. A higher proportion of PHI was found in the coagulopathy group (54%) compared to the no-coagulopathy group (33%) with an OR of 2.4 (95% CI 0.8–8.0). Although the former was non-significant, the observation that a higher proportion of patients died in the coagulopathy group (77%) compared to the no-coagulopathy group (13%) with an OR 23.06 (95% CI 5.5–95.9)) was significant.

In the subgroup of patients with only focal lesions, thirteen (13%) showed a coagulopathy on ED presentation of 99 patients. The relation with future events was almost similar: PHI OR 2.3 (95% CI 0.7–7.3), for surgery OR 2.5 (95% CI 0.7–8.1), admission to the ICU OR 6.4 (95% CI 0.8–52), and mortality OR 20.6 (95% CI 4.9–86).

### Coagulopathy parameters

Patients with coagulopathy at ED presentation had a mean INR of 1.19 (95% CI 1.12–1.26), a mean APTT of 38.7 (32.0–45.3) s and a platelet count of 170 (93–247) × 10^3^/μl. Patients without a coagulopathy had a mean INR of 1.00 (1.00–1.02), APTT of 27.0 (26.4–27.6) s, and a platelet count of 240 (229–250) × 10^3^/μl. In the coagulopathy group, 53.4% demonstrated an elevated APTT, 46.2% had a low platelet count, and an INR > 1.2 of was observed in 15.4% of patients (Table 2). A medical history of hypertension (HT) was found in 24 patients, 23 in the no-coagulopathy group, and one in the coagulopathy group (*p* value 0.15). Patients with HT used diuretics (50%), beta blockers (33%), angiotensin-converting-enzyme inhibitors (21%), angiotensin-II-receptor blockers (13%), calcium channel blockers (8%), or no medication (8%).

### Time course of coagulopathy and association with outcome

**Fig. 2:**
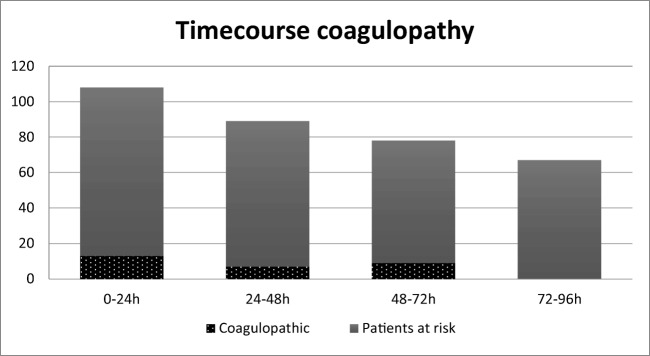
Time course of coagulopathy*. Y*-axis, total patients. The incidence of coagulopathy was 12.0%, 7.9%, 11.5%, and 0% for 0 to 24 h, 24 to 48 h, 48 to 72 h, and 72 to 96 h after admission, respectively (defined as an international normalized ratio (INR) > 1.2 and/or a platelet count below 150 × 10^3^/μl and/or activated partial thromboplastin time (APTT) > 34.5 s)

Patients in the studied cohort often developed a coagulopathy in the days after trauma. Within 96 h after trauma, a total of 29 (27%) patients developed a new coagulopathy. This study found an incidence of coagulopathy of 12.0%, 7.9%, 11.5%, and 0% for 0 to 24 h, 24 to 48 h, 48 to 72 h, and 72 to 96 h after hospital admission respectively (Fig. 2). In fourteen patients, the coagulopathy resolved within 96 h after trauma.

**Fig. 3:**
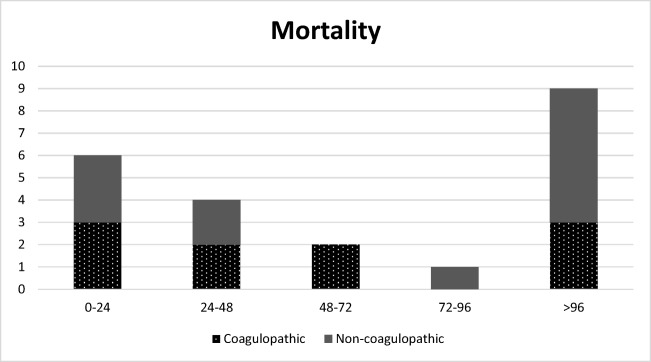
Mortality in cohort. A total of 22 patients died during admission of which ten (45% (odds ratio 23.06 (95% confidence interval 5.5–95.9)) were coagulopathic at presentation to the emergency department

Six patients (three coagulopathic and three non-coagulopathic) died the first 24 h after admission, while four patients (two coagulopathic and two non-coagulopathic) died between 24 and 48 h following hospital admission. Furthermore, two patients (both coagulopathic) died between 48 and 72 h and one patient (non-coagulopathic) died between 72 and 96 h after admission. An additional nine patients (six coagulopathic and three non-coagulopathic) died 96 h after hospital admission. A total of 22 patients died of which ten were coagulopathic upon presentation to the ED (Fig. 3).

## Discussion

In this observational cohort study of 108 patients with an acute t-ICH, thirteen patients (12%) developed a coagulopathy within 24 h after trauma and 22 (20%) patients died within 96 h after admission. The new coagulopathy was not confined to the first 24 h and even occurred at 72 h after trauma. Furthermore, coagulopathy was associated with PHI, surgical intervention, and strongly associated with in-hospital death. Patients who sustained a TBI should therefore be closely monitored because they can develop a coagulopathy and consequently deteriorate in the days following trauma. Although coagulation derailments have been studied previously in the general TBI population, this is one of the first reports showing the occurrence of a coagulopathy in patients who sustained t-ICH [2, 10]. In a recent review by Epstein and colleagues, 22 retrospective and prospective cohort studies were analyzed to examine the incidence and clinical outcome of coagulopathy in the general TBI population [8]. They found a pooled incidence of 35.2% (95% CI 29.0–41.4), which is higher than reported in this study (27%). However, the definition of coagulopathy as reported by this review was not consistent and included different combinations of INR, platelet count, prothrombin time (PT), APTT, fibrinogen, disseminated intravascular coagulation score, modified coagulopathy score, and alpha-2 plasmin inhibitor value. In general, the incidence of coagulopathy after TBI appears to be highly dependable on the used cutoff value and time of blood draw. The current study made use of the INR as a marker for coagulopathy as opposed to a prolonged PT, to avoid interassay variation in PT analyses used by different institutions.

There remains a discrepancy in reports on the time course of coagulopathy. Some studies report an increase of TBI patients who develop a coagulopathy after the presentation at the ED, others report a decrease in coagulopathy incidence [3, 15, 16, 22, 30, 32]. This study found an incidence of around 10% for 24, 48, and 72 h after trauma, indicating that patients with TBI remain at risk for developing a coagulopathy days after the trauma. Only after 72 h, no patients developed a new coagulopathy in this cohort.

This study found a higher proportion of PHI in the coagulopathy group than in the no-coagulopathy group (54% vs. 33%) (OR of 2.4 (95% CI 0.8–8.0)) which is comparable to other studies [1, 18]. Despite the heterogenic definition and cutoff values used, the majority of studies have linked coagulopathy with an increased risk of PHI [1, 7, 18, 20, 26], although some studies report no relation between PHI and coagulopathy (i.e., unchanged additional CT scans in patients with coagulopathy after TBI) [19, 29]. Naturally, part of the PHI is explained by the severity of the primary brain injury more so than by secondary PHI, induced by (unrecognized) coagulopathy.

Finally, this study found a strong relation between coagulopathy and in-hospital mortality. This is similar to recent studies that reported a much higher mortality rate for coagulopathic patients than for patients who did not have a coagulopathy. Moreover, in these studies, the same cutoff value was used to define a coagulopathy (INR > 1.2) [1, 32].

### Strengths and limitations

Most studies that observed a correlation between coagulopathy and TBI focused on the broad TBI population or on specific subgroups based on GSC, time interval, age, or the presence of extracranial injury. This is one of the first studies that identify a coagulopathy in patients with intracranial hemorrhages after TBI. In patients with an intracranial hemorrhage, proper hemostasis is vital to prevent the initial hemorrhage to further expand and cause secondary injury.

Selection bias is an important consideration in this observational study. In the current cohort, the coagulation assessment (INR, APTT, and platelet analysis) was performed following indication by the treating physician, which explains why less information was available on coagulopathy status in the days after presentation. A variation in iatrogenic correction of coagulopathy could also interfere with the time course of coagulopathy. This study did not take correction of coagulation into account. Because it was not in the protocol to have the coagulation status checked the days after trauma, the relationship between clinical outcome (mortality, surgical intervention and PHI) and coagulation status has only been explored for blood drawn at ED presentation.

In the participating hospitals, performing an additional CT scan occurs on indication only. This may lead to biased PHI rates, as neurologically unstable patients are usually indicated for an additional CT scan. Patients with t-ICH who remained clinically stable did not have a follow-up CT scan.

Patients with a coagulopathy within 24 h after trauma presented with a lower mean GCS than patients without a coagulopathy (6.1 vs. 10.9) and thus have a worse initial prognosis. Whether or not the coagulopathy is an independent risk factor and/or possibly (partly) causes intracranial bleeding are topics for future research.

### Future

An important question is whether an intervention exists that can reduce PHI, induced by the coagulopathy after TBI. To tackle this clinical problem, one must find a therapeutic target, constructed from pathophysiological insights. However, coagulopathy after TBI is the result of a complex mechanism that is still largely unknown [25]. Due to the lack of understanding on how TBI induces coagulopathy, no evidence-based clinical intervention is available to prevent or treat the contribution of coagulopathy to the development of secondary injury after TBI.

In the future, the authors will set up a multicenter case-cohort study within the already active Net-QuRe and CENTER-TBI studies to identify differences in specific coagulation parameters between patients with PHI and patients who did not develop secondary hemorrhages [24].

### Conclusion

Coagulopathy after TBI is a complex mechanism that is not fully understood. Patients who sustained an intracranial hemorrhagic injury remained at risk for developing a coagulopathy until 72 h after trauma. Early coagulopathy is associated with PHI, surgical intervention, and increased in-hospital mortality of patients who sustained t-ICH. This indicates that more extensive etiological research with a detailed analysis of the coagulation status after TBI is needed to improve critical care, lower mortality, and improve neurological prognosis for these patients.

## References

[1] Allard CB, Scarpelini S, Rhind SG, Baker AJ, Shek PN, Tien H, Fernando M, Tremblay L, Morrison LJ, Pinto R, Rizoli SB (2009). Abnormal coagulation tests are associated with progression of traumatic intracranial hemorrhage. J Trauma.

[2] Bershad EM, Farhadi S, Suri MFK, Feen ES, Hernandez OH, Selman WR, Suarez JI (2008). Coagulopathy and inhospital deaths in patients with acute subdural hematoma. J Neurosurg.

[3] Carrick MM, Tyroch AH, Youens CA, Handley T (2005). Subsequent development of thrombocytopenia and coagulopathy in moderate and severe head injury: support for serial laboratory examination. J Trauma.

[4] Chang R, Cardenas JC, Wade CE, Holcomb JB (2016). Advances in the understanding of trauma-induced coagulopathy. Blood.

[5] Cohen MJ, Brohi K, Ganter MT, Manley GT, Mackersie RC, Pittet J-F (2007). Early coagulopathy after traumatic brain injury: the role of hypoperfusion and the protein C pathway. J Trauma.

[6] Daabiss M (2011). American Society of Anaesthesiologists physical status classification. Indian J Anaesth.

[7] DeOliveira Manoel AL, Neto AC, Veigas PV, Rizoli S (2015). Traumatic brain injury associated coagulopathy. Neurocrit Care.

[8] Epstein DS, Mitra B, O’Reilly G, Rosenfeld JV, Cameron PA (2014). Acute traumatic coagulopathy in the setting of isolated traumatic brain injury: a systematic review and meta-analysis. Injury.

[9] Finfer SR, Cohen J (2001). Severe traumatic brain injury. Resuscitation.

[10] Folkerson LE, Sloan D, Cotton BA, Holcomb JB, Tomasek JS, Wade CE (2015). Predicting progressive hemorrhagic injury from isolated traumatic brain injury and coagulation. Surgery.

[11] Franschman G, Boer C, Andriessen TMJC, van der Naalt J, Horn J, Haitsma I, Jacobs B, Vos PE (2012). Multicenter evaluation of the course of coagulopathy in patients with isolated traumatic brain injury: relation to CT characteristics and outcome. J Neurotrauma.

[12] Ghajar J (2000). Traumatic brain injury. Lancet.

[13] Giesen PLA, Nemerson Y (2000). Tissue factor on the loose. Semin Thromb Hemost.

[14] Giesen PLA, Rauch U, Bohrmann B, Kling D, Roqué M, Fallon JT, Badimon JJ, Himber J, Riederer MA, Nemerson Y (1999). Blood-borne tissue factor: another view of thrombosis. Proc Natl Acad Sci.

[15] Greuters S, Van Den Berg A, Franschman G, Viersen VA, Beishuizen A, Peerdeman SM, Boer C, ALARM-BLEEDING investigators (2011). Acute and delayed mild coagulopathy are related to outcome in patients with isolated traumatic brain injury. Crit Care.

[16] Halpern CH, Reilly PM, Turtz AR, Stein SC (2008). Traumatic coagulopathy: the effect of brain injury. J Neurotrauma.

[17] Harhangi BS, Kompanje EJO, Leebeek FWG, Maas a IR (2008). Coagulation disorders after traumatic brain injury. Acta Neurochir.

[18] Juratli TA, Zang B, Litz RJ, Sitoci K-H, Aschenbrenner U, Gottschlich B, Daubner D, Schackert G, Sobottka SB (2014). Early hemorrhagic progression of traumatic brain contusions: frequency, correlation with coagulation disorders, and patient outcome: a prospective study. J Neurotrauma.

[19] Kaups KL, Davis JW, Parks SN (2004). Routinely repeated computed tomography after blunt head trauma: does it benefit patients?. J Trauma.

[20] Kurland D, Hong C, Aarabi B, Gerzanich V, Simard JM (2012). Hemorrhagic progression of a contusion after traumatic brain injury: a review. J Neurotrauma.

[21] Laroche M, Kutcher ME, Huang MC, Cohen MJ, Manley GT (2012). Coagulopathy after traumatic brain injury. Neurosurgery.

[22] Lustenberger T, Talving P, Kobayashi L, Barmparas G, Inaba K, Lam L, Branco BC, Demetriades D (2010). Early coagulopathy after isolated severe traumatic brain injury: relationship with hypoperfusion challenged. J Trauma.

[23] Maas AIR, Stocchetti N, Bullock R (2008). Moderate and severe traumatic brain injury in adults. Lancet Neurol.

[24] Maas AIR, Menon DK, Steyerberg EW, CENTER-TBI investigators (2015). Collaborative European neurotrauma effectiveness research in traumatic brain injury (CENTER-TBI): A prospective longitudinal observational study. Neurosurgery.

[25] Maegele M (2013). Coagulopathy after traumatic brain injury: Incidence, pathogenesis, and treatment options. Transfusion..

[26] Oertel M, Kelly DF, McArthur D, Boscardin WJ, Glenn TC, Lee JH, Gravori T, Obukhov D, McBride DQ, Martin NA (2002). Progressive hemorrhage after head trauma: predictors and consequences of the evolving injury. J Neurosurg.

[27] Rezaie AR (2001). Vitronectin functions as a cofactor for rapid inhibition of activated protein C by plasminogen activator inhibitor-1: implications for the mechanism of profibrinolytic action of activated protein C. J Biol Chem.

[28] Roozenbeek B, Maas AIR, Menon DK (2013). Changing patterns in the epidemiology of traumatic brain injury. Nat Rev Neurol.

[29] Stein SC, Spettell C, Young G, Ross SE (1993). Delayed and progressive brain injury in closed-head trauma: radiological demonstration. Neurosurgery.

[30] Stein DM, Dutton RP, Kramer ME, Scalea TM (2009). Reversal of coagulopathy in critically ill patients with traumatic brain injury: recombinant factor VIIa is more cost-effective than plasma. J Trauma.

[31] Talving P, Lustenberger T, Lam L, Inaba K, Mohseni S, Plurad D, Green DJ, Demetriades D (2011). Coagulopathy after isolated severe traumatic brain injury in children. J Trauma.

[32] Wafaisade A, Lefering R, Tjardes T, Wutzler S, Simanski C, Paffrath T, Fischer P, Bouillon B, Maegele M (2010). Acute coagulopathy in isolated blunt traumatic brain injury. Neurocrit Care.

